# A Vision of State Medical Service

**Published:** 1918-11-16

**Authors:** G. T. K. Maurice

**Affiliations:** C.M.G.


### A VISION OF STATE MEDICAL SERVICE.

**By Colonel G. T. K. MAURICE, C.M.G.**—*(concluded from p. 112).*

### Practitioners' Earnings Under State Medical Service.

It has been supposed that, given certain attractions, a man will accept a change of career. Largely it is a matter of economics. If the pay is as good or better than he can get elsewhere, the emoluments more certain, and the life equally pleasant or more so, a man will take new work with little hesitation. What is the pay to be and on what basis will men be likely to drop private practice and take State service gladly and contentedly, feeling they have been fairly treated and therefore ready to work well and cheerfully?

A medical man's earnings are often widely different if expressed in nett instead of in gross, for the expenses of many practices are a high percentage of the takings. There are the income-tax returns as a basis for calculation. These returns express the nett after deduction of expenses. Under a State Medical Service the medical man would be relieved of the expenses of practice, of travelling, of instruments, of consulting-rooms, and of drugs and dressings. In addition to his pay he would receive an allowance for house-rent, which would vary with locality, or an official residence. He would do his rounds in motor-cars provided for the purpose, or would provide his own car and be paid an allowance for it, or, if the nature of his work did not need a car, be provided with other means of travel, or no means at all. Though it be not the truth in every case, none the less, as a rough guide, a man's earnings are an index of his ability and his standing amongst his fellows.

This index, joined with such other factors as common repute, the opinion of his colleagues, and the records of his work, would be a great help to the successful grading of him. Suppose that it were to be found that the average earnings of young men recently qualified and only a year or two in practice were £400 per annum nett, that would seem to be a fair figure on which to ask them to take State service, but from it would be deducted such sum as represented the value of pensions. Such men would be starting in the lowest grade, Grade 1 of the State Medical Service. There would be other men longer in practice, older, more experienced, earning a nett income of, say, £600 a year on an average. They would form Grade 2. Higher again, Grade 3 at £800 a year nett, and so on up to the highest administrative grades, with pay at least equal to the highest ranks of the Civil Service. The actual details of the length of time in a grade, of how far promotion should be by seniority, by the results of examination at the end of a post-graduate course, by recommendation of seniors, are matters to be worked out and evolved. But as flat rates and promotion by seniority are not stimulating to endeavour, special extra rates of pay should be made to especially proficient men. This pay also would be graded. The operating surgeons and consulting physicians of a large London hospital would get a higher grade of special pay than similar appointments in small provincial hospitals, and these again than in the appointments in small cottage hospitals. It is only intended here roughly to outline a scheme. The figures are based on nothing. That is to say, no examination of accounts or income-tax returns has been made, and no attempt to ascertain the real earnings of the most highly successful of the profession. The idea is that, if the Service were to be established in any given year, the young man not long qualified would fall in at a salary, plus house-rent, plus pay of a minor appointment, if given one. The men next senior to them, with due consideration of their previous earnings, their repute and record, would fall in in Grade 2 with a higher salary, house-rent, and the pay of a special appointment if given one. The recognised heads of the profession would fall in in the highest grade of all, and be paid a high salary, a high special pay, and house-rent. The special pay would be for appointments such as operating surgeon, consulting physician, anæsthetist to a district or a large hospital, eye specialist, and so on and so forth, and for administrative appointments. It would possibly be less difficult to work out in detail than it appears to be on first consideration. After the Service was started, year by year retirement under age clauses and death would remove men from near the top, and year by year recruits would come in at the bottom, till in a generation there would be a Service with a steady flow of promotion and a medical *personnel* in various grades making from, say, £300 at the bottom up to, say, £5,000 at the very top. Again it is emphasised that the figures are only names for sums, but the possible prizes to be gained by long and faithful service must be high, for the Service to be efficient must have of the best intellects in the land. Great business concerns are willing to pay thousands a year to their chief managers and organisers, barristers may make tens of thousands, a political career offers enormous money prizes—there are many ways in which able men can reach honour and affluence. So long as this is the case it is vain for the nation to dream that it can get a first-class Medical Service on the cheap.

### Career of Young Practitioner Under State Medical Service.

It may be well to imagine the career of a young man who after qualification enters the State Medical Service. He has done well in the school and has passed his entrance examination with distinction. The reports on him are good. Probably he will, in the first place, be given a post as a junior resident in a large London hospital. He will be a house surgeon. He will get first grade pay and rooms in the hospital, but will pay his own messing and washing. He will do the work of a house surgeon for six months or a year and then be given another appointment such as resident obstetrician in the same hospital or another in some large provincial town. He will have put down his name for certain counties in which he wishes to serve, and when his time as resident is ended he will get orders to report to the chief medical officer of one of the counties he has selected, which depends upon the vacancies occurring. The chief medical officer will order him to report for duty to one of the deputy chief medical officers of his county. He will join up and be appointed for duty at the hospital of a sub-district. Imagine he reports at Trowbridge and is ordered to Swindon division of the county of Wilts. Swindon will be the headquarters of a division and of a deputy chief medical officer and will be a sub

☛ The previous article appeared on November 9, p. 109.

**A Vision of State Medical Service—(*continued*).**

district itself, the hospital and the medical staff serving a larger town and the country places round about. He will be introduced to a considerable staff of colleagues of various grades of service and various lengths of service. The deputy chief will probably attach him to a man in the second or third grade to help work some definite part of the town. His position will have analogies to that of an assistant or a junior partner. He will go of a morning to the medical men's building, which will be near the considerable hospital that will be required for so large a town, and there in the library or other common room meet his colleagues and hear of the work necessary for the day. He will go to one or other of the dressing-rooms or consulting rooms and see some of those who are attending for treatment, and perhaps afterwards go to the hospital to help his associate with an operation. Thereafter the pair of them will arrange the round of visits between them and perhaps he will return to the hospital in the afternoon to give anæsthetics, to do other work or to attend a patient he has sent in. The daily routines will vary with circumstances. He might be attached to the obstetrician of the district or have duties in a special hospital or in the pathological department. It will be a matter of his bent. He would, however, get experience of general practice under an experienced man for his first few years and gradually work up to the next grade. During his time in the second grade he would probably go through a post-graduate course of a few months' duration, during which he would brush up his general medicine and surgery and take up any special branch that appealed to him. We will suppose he chose to take a course in public health and that he passed the prescribed examination in it with honours. He would be eligible for a special billet as a medical officer of health. Such a billet is offered him in Berkshire, and he decides to accept. He finds himself attached to the Reading division with duties as assistant health officer, and his grade pay and a special pay for the special work, in the town of Wokingham. Here he will do a share of the general practice and also the duties of medical officer of health till, if he has shown himself able, he is moved to a public health billet that is of more importance, is full time, and carries a higher rate of special pay. He may decide to continue all his service in public health work and find himself at length the chief public health officer of Birmingham, Liverpool, or other great city, with a high-grade pay, the highest rate of special pay, a handsome income, and a world-wide reputation as an authority on the subject.

Another young man may have a taste and talent for surgery. By similar steps he rises in the Service till he comes to be chief operating surgeon of a large hospital in London, Liverpool, Sheffield, or other great city. His State pay is high, but in addition shall be another chance of prizes material besides the honours and repute he has gained.

It has always been the case that certain surgeons, physicians, and obstetricians have earned great reputations, are in great demand, and are able to command large fees, and always there have been wealthy persons prepared to pay the fees such highly considered men demand for their services. Some have special faith in one, some in another, and some only ask that some one of those of great renown shall operate upon them or those dear to them. There is no reason such system should not continue with a State Medical Service. The State will provide efficient attention for every citizen and will appoint to each his medical attendants and will see to it that each can get the special skill and nursing appropriate to his or her case. Every man will be safe to trust to the organised care offered him, but, if he fancy some special surgeon or physician of whom he has heard, there will be no reason why he should not chose him, if he cares to pay a special rate for that man's services. A rich man, then, has appendicitis. He has at hand in his sub-district a surgeon thoroughly competent to operate on him, and he can, if he choose, take a special ward in his district hospital for the rental charged for it, or he can, if he be frugal minded, use the free wards, and go in and be operated on by the surgeon free of all operation fee. But he has heard that some man has a specially great reputation or is fashionable because he has operated on Royalty, or he fancies him for some reason or no reason. There is nothing to prevent his demanding the services of that surgeon and paying his fee. The fee will vary with the man just as it does now, twenty-five guineas, fifty guineas, a hundred guineas, or more. He arranges for the surgeon of his fancy, has his services and pays his fee. But the surgeon is the paid servant of the State, paid out of taxation or special medical contribution; he cannot therefore be allowed to turn aside from his State Service for special cases and receive all a double pay. He must share his fee with the State. As it is important to stimulate men to make themselves fit for the front ranks of the skilful and to stay there, it would not be politic to take all of such special fees. The great surgeon is drawing £5,000 a year from the State. His special fee is fixed at 100 guineas to 250 guineas. He goes to the rich man, takes 100 guineas fee and gives fifty guineas to the State. So, if he does 100 such special operations in a year he will make £10,000 a year and over for himself, and the State will get back in half-fees all his pay and get work from him into the bargain. By similar arrangements men of less note with no more than local reputations would be open to the choice of the whimsical, or of those that had confidence in them above others. Nor can such a scheme be considered unfair to doctor or patient. To the former ever since he entered the Service the State has assured a competency and given education and opportunity. It is fair the State should share his special gains that come of that opportunity. To the patient the State, through its carefully trained and supervised Medical Service, guarantees competent medical attendance at no more cost than the common taxation necessary to secure it. If through whimsicality or for more excusable reason an individual wishes to upset the State arrangements by a special call, it is not unfair he should pay a special rate for the privilege.

### Subordinate and Unqualified Elements of Service.

So far there has been little consideration of those elements of a Medical Service other than qualified medical men. The other elements will be many and diverse but subordinate. There must be a State Nursing Service to serve the hospitals great and small, and to supply nurses in the homes of the people when need arises. In this branch alone will be great scope for organisation. It is probable that the demand for enrolment would be very large; for the certain pay, the comradeship to be found in the great State nursing institutes, and the certain pension would be very attractive by contrast with the lot of a nurse of to-day. Porters, hospital attendants,

**A Vision of State Medical Service**—*(continued)*.

chemists, motor drivers, sanitary inspectors, massage men and massage women, dispensers, pathology-room assistants, *x*-ray assistants, and many more, all would be absolutely necessary to the vast organisation. For it must be a vast organisation to be effective, and by reason of its vastness there must be decentralisation, or there will be danger of the whole being too unwieldy for efficient working. Each chief medical officer of a county must have much responsibility and much power. Each assistant chief must have much responsibility and much power. The test should be, Is your district run well, is the work done well, are the patients satisfied, is the staff willing at its work and happy in it? If this be not the case the head is at fault and he must go. Go, not out of the Service, but to other work for which he is fitted. Every man has his right niche; it is the business of the administrator to put him in it.

If there ever be a State Medical Service the medical equipment and *personnel* existing when the Service is founded must be utilised, the latter because no other exists, the former because it would be wasteful to discard what is and make entirely new other. So most of the existing hospitals, dispensaries, infirmaries, convalescent homes, and so forth, with much of their contents, would become the property of the State. For the most part these are now held in trust by or are the property of various corporations, and it is perfectly certain that any scheme for their acquisition by the State will meet with bitter opposition. Men will fight as fiercely for patronage as for pelf. But so soon as the State furnishes an efficient Medical Service the need for charitable institutions for treatment will have gone. The charitable members of the community subscribe enormous sums because they feel that, but for the great hospitals and the charity that enables them to do their work, enormous numbers of seriously sick persons would get no treatment worth the name. If, however, the State, by means of a Medical Service and all the necessary buildings and appurtenances thereof, provides thoroughly efficient medical treatment for all classes of the community, the reason for the existence of any hospitals that should have successfully resisted efforts to annex them to the State Service would be gone, the life-giving inflow of charitable subscription would cease, and the institutions would fall into bankrupt decay. There would remain only a few special institutions devoted to treatment limited by fads and dogmas and supported only by fervent faddists and those who have faith in pseudo-scientific formulas. It will be doubtfully wise for the men concerned in the management of a great hospital to set themselves to oppose its becoming the property of the State if the State is determined on organising its own Medical Service.

### Position of Healthy Citizens and Patients Under the Scheme.

ROUGHLY outlined, and with detail purposely left out, here is a scheme, a not impossible scheme, for a great and comprehensive State Medical Service. What will be the position regarding it of the healthy citizen and of the patient under its care?

As very largely, and perhaps chiefly, the work of the Service will be concerned with the prevention of disease and the maintenance of good health, the sanitary matters of country and town will come under it, and this implies the inspection of premises, reports on delinquencies, advice on drainage systems, and other matters, all backed by so strong an authority that even the most obstructive council will be unable to ignore it, food inspection, dairy inspection, suggestions for better water supplies, and absolutely independent men condemning insanitary houses. The inspection of school children, the education of parents and better still of prospective parents, the consideration of building plans, the oversight of dangerous trades and the care of the people engaged in them; all and everything connected with the maintenance of the health of the community and the betterment of the health of the community. It may well happen that eventually the larger portion of the State Medical Service will be engaged in preventative medicine, sanitation, and research, though now the idea conjured by the words "medical man" is one treating the sick rather than one preventing sickness.

The sick man will be the care of a medical man or medical men told off for the care of him by the State. An imaginary sketch will perhaps better convey the actual conditions contemplated than other attempt at detail

John Brown goes to bed feeling seedy. Next morning he feels he is not fit for work. He sends to the hospital of his district by messenger, telegram, or telephone to ask that he may be seen by the doctor. The message is passed to the medical man who visits the village or street in which John Brown lies sick abed. In the course of his round the doctor calls and examines him. If it is an ordinary illness, he prescribes, gives advice, and leaves him till the next morning, when he sees him again. But suppose the doctor finds John Brown very ill indeed, with symptoms that puzzle him and make diagnosis doubtful. He suggests to John Brown that he would be better in hospital. John Brown objects to leaving his house, says his wife will look after him well, and is not at all desirous of going to hospital. As he is not infectious there is no question of compulsion, but the diagnosis is not made and the doctor is anxious. He telephones to the hospital and says he should like a consultation with someone, probably he names the man, either he will be a regularly appointed consultant, as would be the case in a big town, or a general duty medical man well known for his diagnostic acumen and likely soon to be getting a good appointment in a higher grade, and asks if he can meet him at John Brown's house, and, if so, when? The consultation is held. The diagnosis is made; pneumonia with diaphragmatic pleurisy and peritonitis. John Brown is strongly urged to take advantage of the hospital and consents. Would he like the free ward, a small three-bed ward, or a private ward? It is a matter of Do you care to pay? John Brown elects for a three-bed ward. The motor ambulance is telephoned for. It arrives with a nurse and an attendant, and John Brown is run off to the hospital and popped into the bed that is waiting for him. He is seen by the resident, a few notes are taken of his condition on arrival and he is left to the care of the nurses, who will, of course, carry out any treatment ordered. A little later, on return from his round, his original doctor will come in to see him, read the report of the resident, add his own notes on the case to the case sheet, give further directions for treatment, and leave his patient secure in the knowledge that he is under the best possible conditions.

Or John Brown is very ill. He is wasting. He is a bad colour. There is a lump to be felt in his abdomen. Several of the State medical men examine him. The diagnosis is not certain. "Something in the pancreas?" "Perhaps." "Is it malignant?" "There ought to be an exploratory operation." "It will be a very ticklish bit of surgery." "Better he should go to the Central County Hospital." Such are the voices of the consultation. The senior medical man of the district informs the deputy

**A Vision of State Medical Service**—(*continued*).

chief of the division of the case and its nature, and asks for a bed at the Central County Hospital. The deputy chief passes on the request to the chief medical man of the county or direct to the Central Hospital, and is informed that a bed is immediately available or will be available in so many days. John Brown by train or motor ambulance arrives, is seen by the surgeons of the Central Hospital, and such operation as is considered necessary supervised or undertaken by a man specially selected for his skill to be a county operating surgeon and surgical consultant. And so in infinite variety whatever ills human flesh is heir to would be countered and promptly treated by a great organised Service. No delay. No question of pay. No inability to afford the first class, for the first class is in reach of all who really need it.

There is the question of complaints against medical men and individual dissatisfactions. Such go to the chiefs and are investigated. By them wrongs are righted, delinquents censured, and frivolous complaints dismissed. Nothing is perfect, but men and women are human beings, and therefore on the whole anxious to be just and to do rightly. With good will on both sides disagreements between doctors and patients would not be many, and friendships would be made just as they are now.

### Cost and Finances of State Medical Service.

Whoever considers a State Medical Service must consider the cost and how the money to pay for it is to be found. The cost must be a tremendous sum of money. A Medical Service cannot be established efficiently on the cheap. The panel system has taught that lesson. But the actual sum cannot be estimated in such a sketchy outline as is here set forth. Against the cost must be set the present expenditure of each family on doctors' bills, medicine, club subscriptions, hospital subscriptions, Poor-Law and asylum service, public-health work, and so forth. All such expenditure would be absorbed into the one great sum found for an efficient State Medical Service at the command of every individual and every corporate body of the State. The money raised would not be all new money. It is even likely that the total to be raised annually would, by virtue of good organisation, absence of overlapping, and lessened expenses of collection, be substantially less than the present total expenditure on the prevention of disease and the cure of it. But it is no good to try to "do it on the cheap," and it is probable that failure will come to any scheme that does not embrace the whole population from the richest to the poorest. If there be an income limit above which a man does not come under the State Medical Service, that will mean confusion at the dividing line and also that many of the best of medical men will refuse State service to compete for practice amongst the wealthy.

There will, of course, be no legal compulsion under a comprehensive State scheme to force a qualified man into the system. The free lance might exist, but in practice he seldom would be found. The advantage of being in the State service will be such that there will be competition to get in, and those who fail will not be of the capacity to do successful practice, except they adopt fads and the views of peculiar people. A qualified man who fails for or in the State service will usually leave medicine for other than medical work, or emigrate.

The money could be raised in at least three ways. Out of general taxation on the Budget, by rates, or by a special public health assessment applicable to all adults according to their means. The last would probably be the best, for then every man would know for what he was paying and how much he was paying for it. This medical assessment might be on the rent of the house; this sum is now used often in mixed general practice as a guide to charges. It might be a special income tax following an Indian system under which Indian civilians of all classes contract to pay a certain proportion per month of their pay in return for medical attendance. This system is adopted to avoid a large unexpected doctor's bill for some year during which sickness may happen to strike heavily on the family. Other forms of assessment can be devised. It would be a great thing that every man should know and feel he was paying his proportional share and had his attendance and nursing of right and not of charity. He who could not or would not pay his due should be stigmatised a pauper and treated apart from the self-supporting citizen.

Here in the rough is a scheme for a great Service to be highly organised but elastic in detail. The organisation should not have a cast-iron constitution elaborated to suffocation of it, but should be something capable of change, of evolution. There must be centralisation, but the head office should hold the reins lightly and use them little. There must be great freedom allowed to each county head, for the different conditions of counties do not permit of a successful uniformity in details. Make broad rules and trust the man. If he fails he goes, to work he can do. It is useless to try to make rules so nice that they shall keep all in a straight path of uniform excellence. If a man does his job well he will go on to a higher post. If he fails in a high post he will revert to a lower one. If he is false to trust he will meet punishment. If he does well he will get reward. Such offers and the natural emulation of men under the stimulation of a corps in which they take pride and are happy will serve to keep the standard high.

All classes must come under the service for treatment, because no class can possibly avoid the benefits of the sanitary work, the research work, and the preventative work, and treatment cannot be separated from these. But individuals, though they must pay their due share and so will have their right to treatment by the men detailed to treat them, will also have the right to refuse the man appointed and to choose another, provided they are prepared to pay fees in addition to their normal taxation. In practice most will be satisfied with the man supplied, just as now most are satisfied with the doctor who chances to be in their street or near it.

Every sick man, woman, or child will be entitled to the services of a man with skill competent to undertake the case. Every medical man will be free to work undisturbed by anxieties financial, and will have consultants at his call, instruments to his need, hospitals and nurses for the care of his patients if he thinks such necessary, laboratories to aid his diagnosis, and every apparatus of the science of healing within his reach. And, beyond all this, great rewards for his good work and honours to stimulate his ambition. The State collectively guarded and taught and treated will improve in health and with that in wealth. The A1 Empire will not be in danger of being run by C3 men.

This scheme is put forward for consideration and constructive criticism. Whether it commend itself to the medical profession or no State interference is coming, and medical men should be prepared to meet it, not with flat negation and a *non possumus*, for it is vain to kick against the pricks, but with our own plans ready, with organisation for collective bargaining, with patriotic readiness to compromise, and to meet the wishes of the community of which we are a part, and with far-sighted, wide-visioned statesmanship to guide the great new service, if it come into being, for the common good.

